# Meta-Analysis of Rumen-Protected Methionine in Milk Production and Composition of Dairy Cows

**DOI:** 10.3390/ani12121505

**Published:** 2022-06-09

**Authors:** Chunbo Wei, Tao He, Xuanchen Wan, Siwen Liu, Yibo Dong, Yongli Qu

**Affiliations:** Department of Animal Science, College of Animal Science and Veterinary Medicine, Heilongjiang Bayi Agricultural University, Daqing 163316, China; weichunbo@byau.edu.cn (C.W.); ht960704@163.com (T.H.); wxc97513@163.com (X.W.); 13209175776@163.com (S.L.); dybdyb0616@163.com (Y.D.)

**Keywords:** meta-analysis, rumen protected methionine, dairy cattle, milk yield, milk composition

## Abstract

**Simple Summary:**

In terms of amino acid nutrition of dairy cows, many scholars have shown that adding rumen-protected methionine to dairy cow feed can improve milk yield and milk components such as milk protein, lactose and milk fat, but the research of some scholars is inconsistent. This paper aims to summarize and analyze all the research contents through meta-analysis and comprehensively understand the impact of rumen-protected methionine on the milk yield and milk composition of dairy cows. The results show that adding rumen-protected methionine to cow feed did not significantly improve milk yield nor the lactose concentration in milk but did improve the fat and protein concentrations in milk, and the effects were better in the high-protein feed than that in the low-protein feed.

**Abstract:**

This study aims to evaluate the influence of rumen-protected methionine (RPM) on the milk yield and milk compositions of dairy cows by employing a meta-analysis method. The articles in the publication databases between January 2010 and January 2022 which reported on various concentrations of RPM supplements in dairy cow diets and then monitored the milk yield and milk compositions were searched. A total of 14 studies were included, covering 27 treatments with a total of 623 dairy cows. Comprehensive Meta-Analysis V3 was used for statistical analysis, the forest map was drawn by the standard mean difference (SMD) with a 95% confidence interval (95% CI), and the SMD was calculated by a random effect model. The dose effect curve was drawn by fitting the SMD and RPM dose of each study to explore the optimal dosage of RPM. Compared with the basal diet, the RPM supplement significantly increased the percentages of milk fat (SMD (95% CI): 1.017% [0.388, 1.646]) and milk protein (SMD (95% CI): 0.884 [0.392, 1.377]). However, the milk yield (SMD (95% CI): 0.227 kg/d [−0.193, 0.647]) and lactose concentration (SMD (95% CI): 0.240% [−0.540, 1.020]) were not affected. The subgroup analysis found that the effect of the RPM supplement on the milk fat and milk protein was greater in the high-protein feed than in the low-protein feed. Multiple regression analysis showed that feeding RPM significantly improved the milk yield and milk protein percentage of dairy cows. The results of the dose–effect analysis show that the optimal range for the RPM was 7.5–12.5 g/d. RPM supplements in a dairy diet can improve the milk protein percentages and milk fat percentages of dairy cows.

## 1. Introduction

Dairy NRC (2001) pointed out that when legume feed, corn silage, soybean meal, and corn kernel are added to the diet of dairy cows, methionine is one of the main limiting amino acids in dairy cows, and it has a great impact on the milk yield and quality of dairy cows. Microbial methionine synthesis in the rumen is relatively small, and most plant feeds lack this amino acid [1]. However, the dairy NRC (2021) has confirmed that the principle of the amino acid barrel has been broken, and methionine is no longer the limiting amino acid in dairy cow diets. Therefore, whether the first limiting amino acid in dairy cow amino acid nutrition will still be there in the future and whether methionine has an impact on dairy cow milk yield and milk quality is a matter of great concern. However, when methionine was directly added to the diet, its utilization rate was low. This was due to the fermentation and degradation by rumen microorganisms, resulting in the reduced bioavailability of methionine in the small intestine of dairy cows. Therefore, to improve the production potential and quality of dairy products, the methionine was first physically or chemically modified to prevent its degradation in the rumen, becoming known as rumen-protected methionine (RPM) [2].

In recent years, studies have investigated the effects of RPM feeding on the milk yield and compositions of dairy cows. It was found that adding RPM could increase the milk yield and fat percentages of dairy cows [3,4]. However, the results of some studies have shown that RPM does not significantly affect the milk yield or composition [5]. Due to differences in the experimental designs, feeding environments, RPM purity, and cow breed selection, we assume that RPM has a certain impact on the milk yield and milk compositions, but we do not know the extent of its impact. Therefore, it is highly necessary to explore the influence of RPM on the milk yield and milk compositions of dairy cows through meta-analysis.

## 2. Materials and Methods

### 2.1. Retrieval Strategy

We collected the existing literature on rumen-protected methionine in Web of Science, PubMed, ScienceDirect, and other databases from January 2010 to January 2022 and calculated the contents of all metabolic amino acids in the small intestine to determine the optimal amount of RPM. The keywords for our search were as follows: (amino acid OR rumen protected amino acid OR methionine OR rumen protected methionine) AND (Dairy cattle OR Cow OR Cows OR Holstein Cow OR Dairy Cow) AND (Milk OR Milk protein OR milk fat OR Milk protein OR Milk protein percentage OR lactose percentages OR Milk fat percentages).

#### 2.1.1. Inclusion and Exclusion Criteria

The meta-analysis criteria were as follows: (1) literature research on the effect of RPM on milk production and its components (milk protein, milk fat, and lactose), (2) rumen methionine was used in the experiments, and the rumen absorption percentage and effective dose equivalent were provided, and (3) the cows used in the experiment were healthy Holstein cows during lactation. The exclusion criteria were as follows: (1) no basic diet control group was provided for the experimental and control groups, (2) rumen methionine was fed with other substances, (3) no reports were provided on the milk yield, fat, or protein percentages, lactose percentage, or other related indicators, and (4) the feeding animals were in a state of stress or intoxication.

#### 2.1.2. Information and Data Extraction

The following data were extracted from each study: author information (first author, year), RPM feeding dose, percentage of crude protein (CP, %) in the feed, the number of samples in the control and experimental groups (*n*), and the mean and standard deviation (SD) or standard error (SEM) of the milk yield, fat, protein, and lactose percentages after feeding. The subgroups were a high-protein group (CP > 16%) and low-protein group (CP < 16%).

### 2.2. Statistical Analysis

#### 2.2.1. Statistical Software

Comprehensive Meta-Analysis V3 (https://www.meta-analysis.com) (accessed on 1 February 2022) was used for statistical analysis of the included data to evaluate the standardized mean difference (SMD), heterogeneity, and weight of each group [6]. Sensitivity analysis and meta regression analysis were performed by CMA software. In addition, a funnel plot and Egger’s test were used to detect publication bias [7].

#### 2.2.2. Effect and Heterogeneity

The standardized mean difference (SMD) was used to evaluate the milk yield and milk compositions of cows with and without RPM supplementation. Variations among the SMD treatment levels were assessed using the I^2^ statistic [8], which measured the effect of heterogeneity on the meta-analysis. The significance was determined by an χ^2^ test, and the significance level was 0.05.

#### 2.2.3. Weighting

Weighting was performed by the inverse of the variance in a hierarchical effects model that included robust variance estimation [9]. Each variance comparison between the RPM group and control treatments was calculated as the square of the pooled SD [10]. The SD for the RPM group and control for each comparison was calculated from the reported SEM such that

(1) $$\mathrm{SD}=\mathrm{SEM}\times \sqrt{n}$$

where n is the number of experimental units.

#### 2.2.4. Sensitivity Analysis and Meta-Regression

The impact of any single study on the overall results was assessed using leave-one-out sensitivity analysis, in which each study was iteratively removed, and the findings were compared to the overall meta-analysis. If there were still more than 10 data in the excluded indicators, meta-regression analysis would be conducted. Meta-regression analysis was used to identify effects of the covariates (year of publication and test cycle) on the supplement RPM response for the dairy cow, using the SMD as the dependent variable.

#### 2.2.5. Publication Bias and Dose–Response Curve

Publication bias was examined using a funnel plot [11] and Egger’s regression method [12]. When Egger’s *p* < 0.10, it was considered that there was significant publication bias.

By combining the effect value with the corresponding dose, the dose effect curve was fitted by the linear regression method. When the curve rose, it was considered that the breast milk trait was improved at this dose.

## 3. Results

### 3.1. Included Studies

A total of 995 related studies were retrieved from all databases using the above retrieval methods. The retrieved studies were screened according to the corresponding inclusion criteria. After excluding the duplicate and unqualified literature, the meta-analysis included 14 studies, as shown in Table 1 [1,2,5,13,14,15,16,17,18,19,20,21,22,23]. To make it easier to visualize the studied datasets, the mean, maximum, minimum, and standard deviation of each study parameter are summarized in Table A1 of Appendix A.

### 3.2. Publication Deviation Analysis

The publication deviation of the literature was tested by Egger’s regression analysis. The results are shown in Table 2. There was no significant heterogeneity in the four traits (milk yield, milk fat, milk protein, and lactose) for the tested cows (*p* > 0.10).

### 3.3. The Effect of RPM on the Milk Yield of Dairy Cows

**Figure 1:**
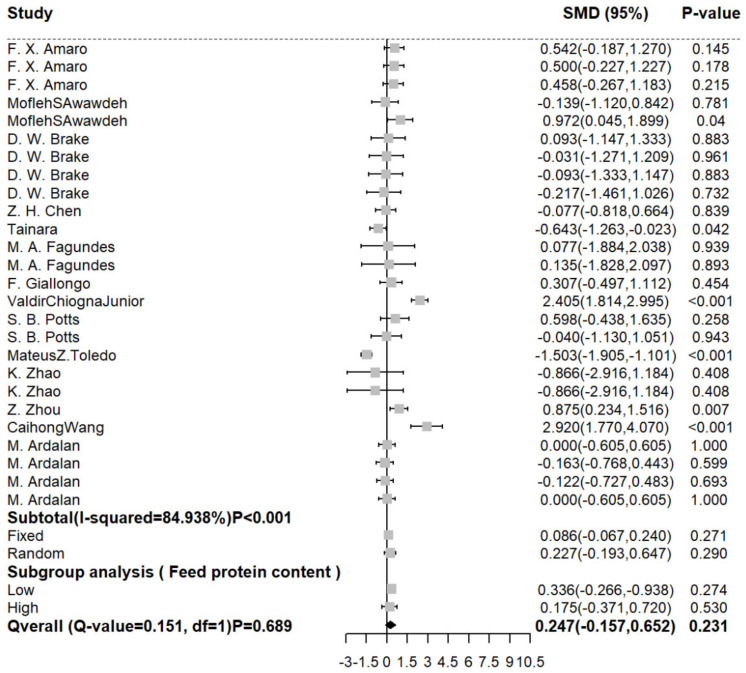
Forest diagram of meta−analysis of RPM on milk yield of dairy cows. The black solid line represents the average difference of the effect, the point on the left of the black solid line represents the decrease of the effect, the point on the right represents the increase of the effect, the grey square represents the effect value of each study group, and the black square represents the overall effect value.

Compared with a basic diet without RPM, adding RPM to the cow diet did not improve the milk yield (SMD (95% CI): 0.227 kg/d [−0.193, 0.647]) significantly (shown in Figure 1). In terms of heterogeneity, I2 (84.938%) showed significant heterogeneity (*p* < 0.001). In order to reduce the heterogeneity, high- and low-protein feed were used for subgroup analysis. The subgroup analysis showed that whether high- or low-protein feed was used, it would not affect the increase in milk yield. At the same time, after the subgroup analysis, it was found that the heterogeneity decreased significantly. Therefore, it can also be judged that the difference between a high-protein diet and a low-protein diet for cows may be one of the sources of heterogeneity.

### 3.4. The Effects of RPM on the Milk Compositions of Dairy Cows

**Figure 2:**
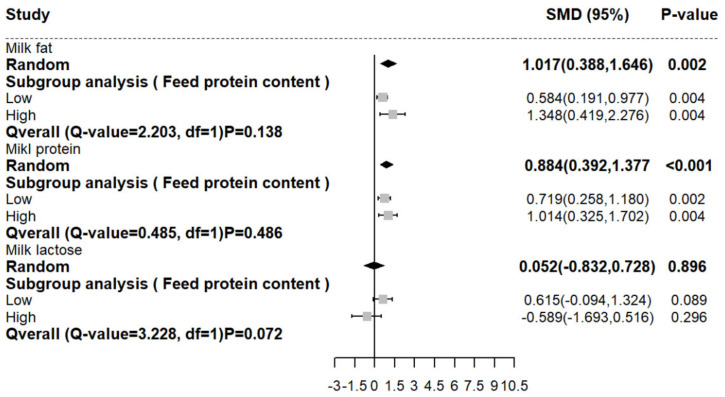
Forest diagram of meta−analysis of RPM’s effect on milk compositions of dairy cows. The black solid line represents the average difference of the effect, the point on the left of the black solid line represents the decrease of the effect, the point on the right represents the increase of the effect, the grey square represents the effect value of each study group, and the black square represents the overall effect value.

The addition of RPM to the diet significantly improved the fat (SMD (95% CI): 1.017 [0.388, 1.646]) and milk protein (SMD (95% CI): 0.884 [0.392, 1.377]) contents compared with those of the basic diet. However, the lactose percentages (SMD (95% CI): 0.052 [−0.832, 0.728]) were not significant compared with those of the basic diet (Figure 2). Significant heterogeneity (*p* < 0.05) was detected by the heterogeneity test of the three indica-tors for milk composition. Subgroup analysis showed that RPM had a greater effect on the high-CP feed in terms of milk fat and milk protein.

### 3.5. Sensitivity Analysis and Meta-Regression Analysis

After sensitivity analysis, no studies were found to cause a large shift in the overall effect size. Further meta-regression analysis was carried out on indicators containing more than 10 groups of data. As seen in Table 3, a different test cycle and publication year had no significant effect on the milk fat or lactose percentages (*p* > 0.05). However, the milk yield and milk protein percentages showed a significant (*p* = 0.002 and *p* = 0.038, respectively) correlation with the reported test cycle (Table 3).

### 3.6. Prediction of Optimum Addition of RPM

**Figure 3:**
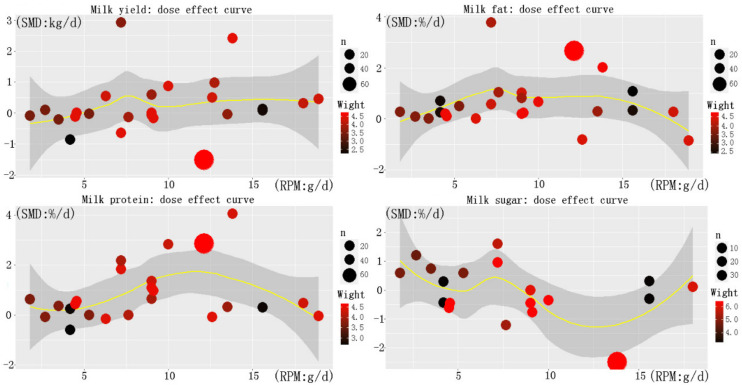
Fitting effect quantity bubble diagram. The above four subgraphs are dose fitting effect diagrams of milk yield, milk fat percentage, milk protein percentage and lactose percentage respectively. The dose curve in the figure is drawn with the included RPM study level as the abscissa and the corresponding SMD as the ordinate, where the size of the point represents the weight and the color represents the sample size (n) of the study sample size. The curve is obtained by nonlinear method.

According to the skewness kurtosis test method in Table A2 it was proven that the data had a normal distribution, so the dose effect curve could be fitted. The dose–effect relationship curves of the RPM and protein, fat, and lactose percentages are shown in Figure 3. The fitting curves shown are relatively smooth and have fluctuation trends. According to the upward and downward trends of the curves, we found that the optimal dosage range in the dose–effect curves of the milk yield and milk fat was between 5 and 10 g/d. However, according to the results of this meta-analysis, it can be found that the addition of RPM to the diet of the dairy cows had the greatest impact on the milk protein in each milk trait, while the results of the dose–effect curves of the milk protein show that the optimal addition amount is between 10 and 15 g/d. After full consideration, the relatively optimal dosage of RPM may be in the range of 7.5–12.5 g/d. If we wanted to further determine the optimal dosage, we needed to carry out gradient dose tests in this range.

## 4. Discussion

Through the study of a large amount of publications, it was found that most scholars did not show the metabolizable amino acids that eventually reach the rumen in the text but defined the content of rumen-protective amino acids directly added to the feed. However, because all kinds of added rumen-protective amino acids are not produced in the same company, and the rumen passing percentage is different, the metabolizable amino acids that eventually reach the rumen are different. Therefore, in the process of extracting RPM content data, this paper calculated the content of metabolizable amino acids that eventually reach the small intestine. The difference between adding different RPM contents could be ignored. The results showed that adding RPM to the diet did not significantly improve the milk yield of the dairy cows, which was consistent with the research results of some scholars [2,5], Although the dairy NRC (2001) pointed out that adding RPL or RPM can improve milk yield, which is likely to be achieved by improving the efficiency of the MP in milk protein synthesis, there are also reports that adding methionine + lysine can improve the milk yield more than providing only one AA [24,25,26]. As for the results for the milk composition index, adding RPM to the diets of dairy cows can significantly improve the milk fat rate and milk protein rate. Through subgroup analysis, it can be found that the effect of adding RPM to high-protein feed on the milk fat percentage and milk protein percentage is significantly greater than that of adding it to low-protein feed, indicating that adding RPM does not meet the demand of low-protein feed rather than high-protein feed. There are many studies on the effect of RPM on the milk fat percentage of dairy cows, although the results are inconsistent. In previous reports, Wang et al. [27] and others found that the milk fat percentage of Holstein cows decreased in the middle of lactation after adding RPM, while Wang et al. [28] and others found the opposite. Milk fat is one of the most unstable components in milk, as it is easily affected by dairy cow varieties, physiological states, ration ratios, seasons, diseases, or other factors. Generally speaking, the milk fat percentage is low in summer and tends to increase in winter, while mastitis and rumen acidosis will also lead to a decline in milk fat. Some scholars have proven that methionine may play dual roles in the mechanism of fat content increases. First, the increased availability of methionine may facilitate the synthesis of choline and transport of lipids as extremely low-density lipoproteins. These changes may prevent accumulation of fat in the liver and increase lipid availability to mammary tissue. Second, in addition to its role as a precursor for protein synthesis, by donating a methyl group in trans-methylation reactions, methionine may contribute to de novo lipid biosynthesis, thus increasing the fat content in milk [29].

In a study by Weekes et al. [30], methionine, as a limiting amino acid in cow nutrition, could affect the lactation process of cows. Adding RPM to the diet could reduce the degradation of methionine by rumen microbes and increase the content of metabolizable amino acids reaching the small intestine to improve the absorption efficiency of methionine and promote milk protein synthesis. Lactose is very stable in milk ingredients and is not sensitive to other factors. In this study, most reported that rumen methionine did not affect the lactose percentage in milk, which is consistent with our analysis implications.

In the research on rumen-protective amino acids, most works focused on the principle of the first restrictive amino acid proposed by the dairy NRC (2001). However, according to the latest report of the dairy NRC (2021), whether the so-called first restrictive amino acid still exists when the amino acid barrel principle is broken is a worthwhile problem to explore. According to the research results of this paper, the addition of rumen-protected methionine to the diet of dairy cows can affect the milk fat and milk protein contents of the dairy cows. At the same time, the crude protein content in different diets of dairy cows also affects the effect of adding RPM. Many scholars have also proven that the effect of the combination of lysine and methionine is greater than that of adding AA alone [24]. Therefore, in this paper, the authors believe that the first limiting amino acid affecting the amino acid nutrition of dairy cows still exists. In future research, we can try to add different rumen-protected amino acids together so as to select the best first restrictive amino acid according to different conditions in the production process.

## 5. Conclusions

The results show that adding RPM to the diet of dairy cows could significantly improve the fat and protein contents of milk from dairy cows. Egger’s method showed that all results were stable and reliable, and there was no publication offset. The optimal dose range for metabolizable methionine was 7.5–12.5 g/d. The meta-analysis combined the relevant literature from January 2010 to January 2022 and provided some guidance for the production practice and research direction of rumen-protected methionine at a statistical level.

## Figures and Tables

**Table 1:** Details of the included literature.

Author	Publication Year	Number of Cows	Included Indicators	PRMet Content
Amaro	2022	60	Milk yield, fat, protein	0 g/d, 6.3 g/d, 12.6 g/d, 18.9 g/d
Awawdeh	2016	32	Milk yield, fat, protein, lactose	0 g/d, 7.65 g/d, 12.75 g/d
Ardalan	2021	104	Milk yield, fat, protein, lactose	0 g/d, 4.56 g/d, 9.12 g/d, 4.5 g/d, 9 g/d
Brake	2013	25	Milk yield, fat, protein, lactose	0 g/d, 2.7 g/d, 5.3 g/d, 1.8 g/d, 3.5 g/d
Wang	2020	24	Milk yield, fat, protein, lactose	0 g/d, 7.2 g/d
Chen	2011	28	Milk yield, fat, protein, lactose	0 g/d, 9 g/d
Fagundes	2018	8	Milk yield, fat, protein, lactose	0 g/d, 15.6 g/d
Giallongo	2016	24	Milk yield, fat, protein, lactose	0 g/d, 18 g/d
Junior	2021	76	Milk yield, fat, protein, lactose	0 g/d, 13.8 g/d
Michelotti	2021	42	Milk yield, fat, protein, lactose	0 g/d, 7.2 g/d
Potts	2020	28	Milk yield, fat, protein	0 g/d, 9 g/d, 13.5 g/d
Toledo	2017	122	Milk yield, fat, protein	0 g/d, 12.1 g/d
Zhao	2019	6	Milk yield, fat, protein, lactose	0 g/d, 4.19 g/d
Zhou	2016	41	Milk yield, fat, protein, lactose	0 g/d, 10 g/d

**Table 2:** Egger’ s bias detection results for the publications.

Items	*p*_Egger_^1^
Milk yield	0.253
Milk fat	0.303
Milk protein	0.164
Milk lactose	0.368

^1^*p* value of Egeer’s test with publication year as covariate in meta-regression analysis.

**Table 3:** Results of regression analysis for covariates.

Items	Publication Year ^1^	Test Cycle ^2^
Milk yield	0.556	0.002 *
Milk fat	0.807	0.540
Milk protein	0.685	0.038 *
Milk lactose	0.698	0.161

^1^*p* value of *t*-test with publication year as the covariate in meta-regression analysis. ^2^ *p* value of *t*-test with test duration as the covariate in meta-regression analysis. * *p* < 0.05.

## Data Availability

The datasets used and analyzed during the current study are available from the corresponding author on reasonable request.

## Appendix A

**Table A1:** Descriptive statistics of data in the literature.

	Mean		Maximum		Minimum		Standard Deviation	
	Control	Met	Control	Met	Control	Met	Control	Met
Milk yield	38.5	38.7	46.4	46.4	23.3	23.2	1.35	1.35
Milk fat	3.4	3.5	4.0	4.2	2.8	2.8	0.06	0.08
Milk protein	3.0	3.1	3.6	3.5	2.77	2.81	0.49	0.49
Milk lactose	4.9	4.9	5.23	5.25	4.6	4.54	0.04	0.04

**Table A2:** SMD descriptive statistics and normal distribution test.

Items	Mean ^1^	Kurtosis ^2^
Milk yield	0.179	3.678
Milk fat	0.614	3.351
Milk protein	0.817	3.817
Milk lactose	−0.0376	3.439

^1^ Average value of SMD. ^2^ Skewness kurtosis test in normal distribution test. If kurtosis is >3, the data have a normal distribution. If the kurtosis <3, then the opposite is true.
